# ON–OFF nanopores for optical control of transmembrane ionic communication

**DOI:** 10.1038/s41565-024-01823-x

**Published:** 2025-01-21

**Authors:** Xingzao Wang, Aidan Kerckhoffs, Jorin Riexinger, Matthew Cornall, Matthew J. Langton, Hagan Bayley, Yujia Qing

**Affiliations:** Department of Chemistry, University of Oxford, Oxford, UK

**Keywords:** Nanopores, Nanopores, Photochemistry

## Abstract

Nanoscale photoswitchable proteins could facilitate precise spatiotemporal control of transmembrane communication and support studies in synthetic biology, neuroscience and bioelectronics. Here, through covalent modification of the α-haemolysin protein pore with arylazopyrazole photoswitches, we produced ‘photopores’ that transition between iontronic resistor and diode modes in response to irradiation at orthogonal wavelengths. In the diode mode, a low-leak OFF-state nanopore exhibits a reversible increase in unitary conductance of more than 20-fold upon irradiation at 365 nm. A rectification ratio of >5 was achieved with photopores in the diode state by either direct or alternating voltage input. Unlike conventional electronic phototransistors with intensity-dependent photoelectric responses, the photopores regulated current output solely based on the wavelength(s) of monochromatic or dual-wavelength irradiation. Dual-wavelength irradiation at various relative intensities allowed graded adjustment of the photopore conductance. By using these properties, photonic signals encoding text or graphic messages were converted into ionic signals, highlighting the potential applications of photopores as components of smart devices in synthetic biology.

## Abstract (web summary)

Here the authors present photo-nanopores in which α-haemolysin monomers are modified with arylazopyrazoles, allowing quantitative photoswitching of the transmembrane ionic current, analogous to electronic diodes and light-tunable resistors in response to light.

## Main

The remote control of transmembrane ionic communication can be facilitated by various molecular tools that respond to external stimuli (for example, light^1–5^, temperature^6^, magnetic^7^ or electrical fields^8^). Among them, optically activated devices fitted with photoresponsive functional groups that leverage light inputs to achieve spatiotemporal modulation of ionic signalling across membranes have garnered attention^9–11^.

In top-down synthetic biology, ion fluxes across cell membranes can be modulated by nanodevices containing photoswitchable chromophores. For example, natural light-driven ion transporters, such as microbial rhodopsins^12,13^, generate or dissipate electrochemical gradients through chromophore isomerization. This enables regulation of the electrical activities of rhodopsin-expressing cells, thereby manipulating basic biological activities such as calcium signalling^14,15^ and neuronal firing^16,17^. These optogenetic tools can also function as integral parts of miniaturized bioelectronic systems^18,19^. Optical control can also be introduced into natural ion channels through the use of synthetic photoisomerizable allosteric ligands that regulate the opening (ON) and closing (OFF) of ligand-gated receptors, such as ionotropic glutamate receptors^20^, transient receptor potential channels^21^ and voltage-gated K^+^ or Ca^2+^ channels^5,22^.

In bottom-up synthetic biology, to modulate transmembrane ionic signalling, there is a high demand for photoresponsive devices featuring modular design, ease of assembly, immediate and reversible ON–OFF responses, sustained ON and complete OFF states and the potential to respond to additional input stimuli. Fully synthetic systems offer broad design and application scopes, but face challenges in affording efficient, real-time and light-actuated ion movements across membranes. For example, DNA nanopores equipped with a photoisomerizable lid strand were optically activated before inserting into lipid bilayers^23^ and synthetic ion transporters with built-in photoswitches moved ions down a transmembrane gradient one at a time^24,25^. Semi-synthetic approaches are also pursued to leverage the natural ON–OFF machinery of protein channels. Chemical engineering of proteins with photoswitches enabled the transient light-activated opening of mechanosensitive channels^4^ or the timed irreversible formation of protein nanopores^26,27^. New designs are required to achieve prolonged activation and reversible ionic responses to light.

Here, we describe reversible ON–OFF photopores, which exhibit a long open lifetime in the ON state and a low background current in the OFF state. The ON–OFF machinery was engineered into a protein construct not originally evolved to open and collapse, and can be activated in real time when embedded in membranes. This was achieved by covalent modification of α-haemolysin (αHL) monomers with arylazopyrazoles that exhibit almost quantitative photoswitching between *E* and *Z* isomers and high thermal stability in both states. The photopores were characterized at both the single-molecule and ensemble levels to showcase their ability to permit or restrict transmembrane ionic current in response to light. The pores exhibit behaviours analogous to both electronic diodes (that is, one-way current flow) and light-tunable resistors (that is, current modulation) depending on the irradiation wavelength. The properties of the photopores were exploited to generate light-to-ionic signal conversion.

## Construction of photoreversible nanopores

**Fig. 1:**
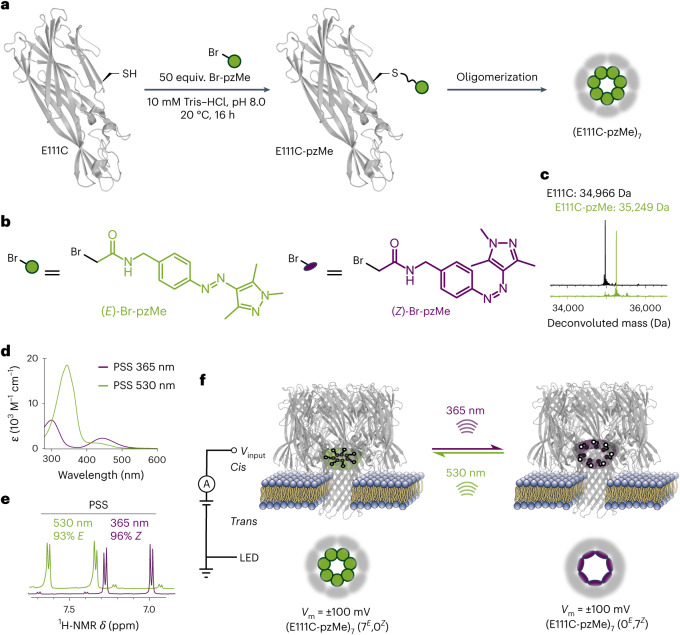
Construction and characterization of the (E111C-pzMe)_7_ photopore. **a**, The αHL monomer containing a cysteine at position 111 was modified with (*E*)-bromoacetyl arylazopyrazole (Br-pzMe) before oligomerization to form the (E111C-pzMe)_7_ photopore. **b**, Br-pzMe exists either as the *E* isomer (green) or the *Z* isomer (purple). **c**, The deconvoluted masses of the E111C and E111C-pzMe monomers, confirming the quantitative modification of the αHL monomer with pzMe. **d**, UV–vis spectra of *E* isomer (green) or *Z* isomer (purple) of Br-pzMe in DMSO at room temperature. **e**, The PSS of Br-pzMe examined by the ^1^H NMR chemical shifts of the aromatic protons in DMSO-*d*6. **f**, The modulation of ionic current passing through single or multiple photopores was evaluated by using voltage-clamp electrical recording, in which a positive transmembrane potential (*V*_m_) was defined as one in which positive charge moved through the bilayer from the *trans* side of the pore to the *cis* side. LED light was collimated and projected onto the bilayer from the *trans* side. Irradiation at 530 nm or 365 nm isomerized arylazopyrazole groups within pores, thereby populating photopores in either predominantly *E* or *Z* states. When all seven arylazopyrazoles adopted the *E* or *Z* configuration, such configurations were named as (7^*E*^, 0^*Z*^) or (0^*E*^, 7^*Z*^). Source data

To construct the photopores, we functionalized αHL, a heptameric protein nanopore, with photoisomerizable chemical groups on the interior of the channel (Fig. 1). The αHL monomers containing a single cysteine residue at position 111, 115, 125 or 129 were separately modified with one of the three types of photoswitches—*o*-fluoroazobenzene (fAzo)^28^, arylazopyrazole (pzH)^29^ or methyl arylazopyrazole (pzMe)^30^—by using thiol-specific chemistry (Fig. 1a,b and Supplementary Figs. 1 and 2). Upon assembly into heptamers, the side chains of these residues project into the lumen of the transmembrane β barrel^31^ (Supplementary Fig. 3). The modification of αHL monomers with one molecule of either fAzo, pzH or pzMe was confirmed by liquid chromatography–mass spectrometry (Fig. 1c and Supplementary Figs. 4 and 5). Modified monomers self-assembled to form homoheptameric pores when introduced into a synthetic lipid bilayer consisting of 1,2-diphytanoyl-*sn*-glycero-3-phosphocholine or in the presence of sodium deoxycholate (Fig. 1a).

The fAzo, pzH and pzMe photoswitches were reported to exhibit high *E*/*Z* or *Z*/*E* isomer ratios at photostationary states (PSSs) and thermal stability in the *Z* state^28,29^. As determined by ultraviolet–visible (UV–vis) and proton nuclear magnetic resonance (^1^H NMR) spectroscopy, the bromoacetamide derivative of pzMe (Br-pzMe) demonstrated the highest isomer ratios at PSSs generated by ‘orthogonal’ wavelengths (Fig. 1d,e). Upon light-emitting diode (LED) irradiation at 365 nm, Br-pzMe was converted primarily to the *Z* isomer (PSS_365_ of 96% *Z*), while when irradiated at 530 nm, Br-pzMe was present predominantly as the *E* isomer (PSS_530_ of 93% *E*) (Fig. 1e and Supplementary Fig. 6). By contrast, Br-fAzo was 85% in the *E* form at PSS_405_ and 91% in the *Z* form at PSS_530_, while Br-pzH was 65% *E* at PSS_365_ and 85% *Z* at PSS_530_ (Supplementary Fig. 6). High isomer ratios were expected to produce tight control of ionic communication through modified nanopores.

## Reversible ON–OFF photopores

The photoreversible modulation of ionic current passing through αHL nanopores containing fAzo, pzH or pzMe photoswitches was first characterized by ensemble electrical recordings in planar lipid bilayers, that is, in bilayers containing hundreds or thousands of pores. A bespoke poly(methyl methacrylate) chamber was made, equipped with a collimated fibre-coupled LED for electrical recording under illumination over a range of intensities from the grounded side (*trans*) of the bilayer (Fig. 1f and Supplementary Fig. 7). To align with the convention of reporting current through αHL pores, we defined a positive current as one in which positive ions moved through the bilayer from the *trans* side of the pore to the *cis* side (that is, from the bottom of the barrel towards the vestibule). The transmembrane potential (*V*_m_) was given as the potential on the *trans* side relative to the *cis* side.

**Fig. 2:**
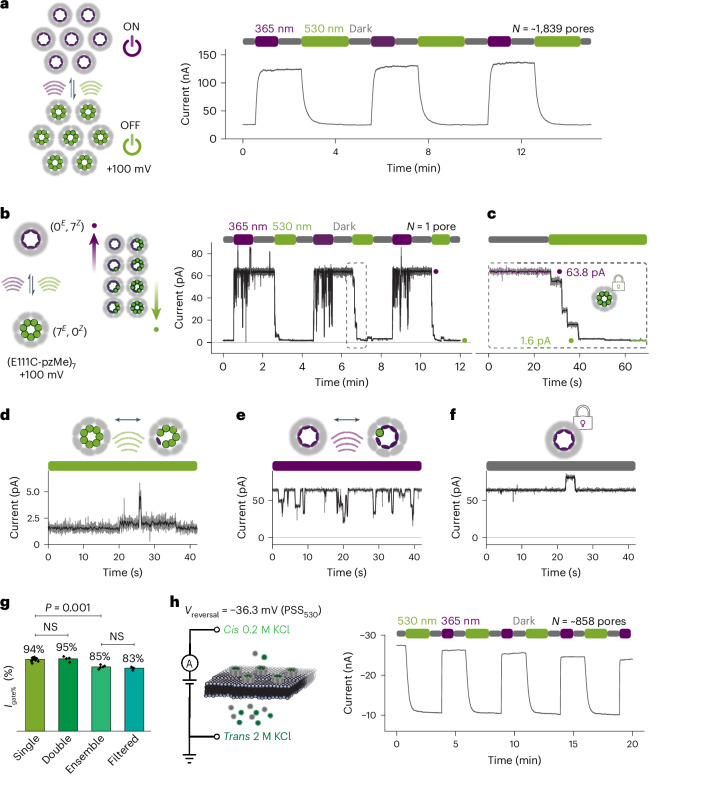
Reversible ON–OFF switching of the (E111C-pzMe)_7_ photopore. **a**, Optical control of the ionic flow through an ensemble of ~1,839 photopores. Irradiation at 365 nm (purple) converted photopores to an ON state, while irradiation at 530 nm (green) produced an OFF state. The current trace demonstrates ON–OFF switching of the photopore ensemble effected by cycles of UV (365 nm) → dark → green (530 nm) → dark. **b**, Optical control through a single photopore switching between (0^*E*^, 7^*Z*^) and (7^*E*^, 0^*Z*^) states. The same light cycle as in **a** was applied. The moving average of the ionic current (grey) is shown in black, which was smoothed with a Savitzky–Golay filter (400 ms window length). During 365 nm irradiation, the photopore switched constantly, causing the black downwards events. By contrast, in the dark, these events stopped. **c**, A zoom-in of the dashed-line box in **b**. Each stepwise decrease in the current was attributed to the isomerization of one or more pzMe photoswitches. **d**,**e**, The ionic current under continuous irradiation, in which the current fluctuated due to switching between the (7^*E*^, 0^*Z*^) and neighbouring (6^*E*^, 1^*Z*^) states at 530 nm (**d**) or between the (0^*E*^, 7^*Z*^) and neighbouring (1^*E*^, 6^*Z*^) states at 365 nm (**e**). **f**, The ionic current in the dark. The current remained stable with occasional upwards bursts of seconds in duration. **g**, The mean per cent gated current (*I*_gate%_) in bilayers containing one pore (*N* = 11 separate pores), two pores (*N* = 4 experiments) or an ensemble of pores (*N* = 4 experiments). Two tailed *t*-tests were performed among the *I*_gate%_ values. In-line filters (355 nm filter for 365 nm LED and 532 nm filter for 530 nm LED) were applied to an ensemble of pores with little effect on *I*_gate%_. **h**, ON–OFF switching of an ensemble of photopores under the electrochemical driving force generated by an asymmetrical KCl concentration across the membrane. The current traces were recorded at +100 mV with an exception of **h** at 25 kHz sampling frequency and filtered using a 5 kHz in-line Bessel filter and a 20 Hz digital Bessel filter. The current trace in **h** was recorded in the absence of an externally applied transmembrane potential. Recording conditions: 2 M KCl (**a**–**f**), 0.2 M KCl (*cis*)/2 M KCl (*trans*) (**h**), 10 mM Tris–HCl, 0.1 mM EDTA, pH 8.5, 24 ± 1 °C. The number of pores in ensemble experiments was estimated, assuming each (0^*E*^, 7^*Z*^) pore contributed +64 pA at +100 mV. Source data.

Interconversion between a high-current level (*I*_ON_) and a low-current level (*I*_OFF_) was recorded at +100 mV with pores equipped with fAzo, pzH or pzMe under alternating wavelengths (365 nm/530 nm for pzH and pzMe, or 405 nm/530 nm for fAzo) (Fig. 2a and Supplementary Figs. 1 and 2). We refer to the lower-current state as the OFF state, in which the ionic flow is partially or almost completely restricted, in comparison with the highest obtainable current level, which is the ON state. In general, the photoreversible nanopores switched to the ON state after irradiation at the shorter wavelength (photoswitches in the *Z* state) and to the OFF state following irradiation at the longer wavelength (photoswitches in the *E* state).

Of the homoheptamers modified at position 111 with fAzo, pzH or pzMe, (E111C-pzMe)_7_ produced the largest per cent gated current (*I*_gate%_ = (1 − *I*_OFF_/I_ON_) × 100%) of 85% at the ensemble level, whereas the other two photoswitches produced low *I*_gate%_ values of ~20% (Supplementary Fig. 1). When the pzMe modification was moved to positions 115, 125 or 129, a decreased *I*_gate%_ was recorded. Without further investigating the mechanism underlying the position- and photoswitch-dependent magnitude of current gating, we proceeded to characterize the (E111C-pzMe)_7_ photopore and explore its applications.

We investigated the photochemistry underlying the ON–OFF behaviour of (E111C-pzMe)_7_ photopores at the single-channel level (Fig. 2b–f). Sequential insertions of pre-formed heptamers, pre-irradiated at 365 nm, revealed that the ON-state conductance of individual photopores ranged from 0.12 to 1.27 nS. The average conductance of a pore (0.62 ± 0.30 nS, *N* = 42 pores) was three times smaller than the wild-type αHL pore (1.81 ± 0.17 nS, *N* = 17 pores) (Supplementary Figs. 8 and 9). An *I*_gate%_ of 95 ± 2.5% (*N* = 21) was consistently recorded at the single-channel level across all the (E111C-pzMe)_7_ photopores (Supplementary Figs. 10 and 11). Reversible photogating was observed by voltage-clamping individual single photopores and exposing them to ten repeats of UV (365 nm) → dark → green (530 nm) → dark (Fig. 2b and Supplementary Fig. 12). Upon UV irradiation, a photopore switched from ON to OFF with a mean response time of 1.8 ± 0.8 s (*N* = 9 transitions), and the OFF-to-ON transition occurred on average within 31.4 ± 7.7 s (*N* = 9 transitions) under the green light. The LED irradiance values (365 nm: 36.2 mW cm^−2^ and 530 nm: 30.3 mW cm^−2^) were derived from measurements of light intensities through a 300-µm-diameter pinhole in place of the bilayer (Supplementary Fig. 7).

The intermediate current levels between *I*_ON_ and *I*_OFF_ were probably produced by the stepwise isomerization of individual pzMe molecules (Fig. 2c). Based on the number of pzMe switches in the *E* or *Z* state within a photopore, we designated the configuration of an ON-state photopore as (0^*E*^, 7^*Z*^), the photopore at the maximum OFF state as (7^*E*^, 0^*Z*^) and a partially OFF pore as one ranging from (1^*E*^, 6^*Z*^) to (6^*E*^, 1^*Z*^) (Fig. 2b). Notably, the intermediate current levels recorded with a single photopore during its ON-to-OFF or OFF-to-ON transitions differed from one transition to another. For example, during the stepwise reduction of the current recorded through an (E111C-pzMe)_7_ photopore under 530 nm irradiation, over ten distinct current levels were recorded across multiple transitions (Supplementary Fig. 13a). The complexity was attributed to both the combinatorial possibilities of the *E*/*Z* configurations of seven photoswitches^32^ and the possible conformations of the photoswitches. For example, in the (5^*E*^, 2^*Z*^) state, there are three possible arrangements of the two *Z*-state pzMe photoswitches around the central axis of the pore (Supplementary Fig. 13b). Once a single photopore reached a terminal ON or OFF state, the current level exhibited fluctuations under continuous irradiation, while remaining relatively stable in the dark (Fig. 2d–f). This was expected as continuous irradiation would drive the reversible isomerization of photoswitches within a photopore: for example, switching between (0^*E*^, 7^*Z*^) and (1^*E*^, 6^*Z*^) states under 365 nm or (7^*E*^, 0^*Z*^) and (6^*E*^, 1^*Z*^) states under 530 nm. In the dark, (E111C-pzMe)_7_ was locked in a single configuration, and no *Z*-to-*E* thermal relaxation was observed for over 1 h after irradiation ceased (Supplementary Fig. 14).

The behaviour of the (E111C-pzMe)_7_ photopores at the ensemble level was compared with that at the single-channel level (Fig. 2g). The *I*_gate%_ value was ~10% lower with multiple pores compared with that of a single pore. This was probably caused by the presence of photopores in partially ON configurations as predicted by the single-channel photoswitching kinetics under continuous irradiation (Supplementary Fig. 15). The incorporation of in-line bandpass filters to reduce the LED bandwidths to 10 nm did not improve the *I*_gate%_ value. By employing an asymmetric KCl concentration across a membrane to establish a physiologically relevant membrane potential (Supplementary Fig. 16), the photoswitching behaviour was reproduced without an externally applied transmembrane potential (Fig. 2h).

## Switchable iontronic diode properties of (E111C-pzMe)_7_

**Fig. 3:**
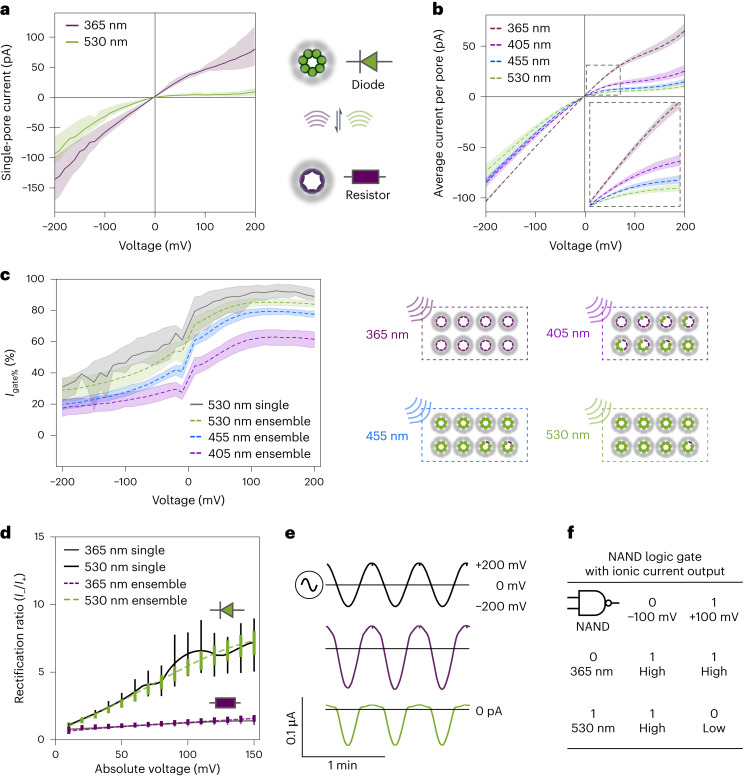
Diode properties of (E111C-pzMe)_7_ photopore. **a**, The current–voltage (*I*–*V*) curves of a single (E111C-pzMe)_7_ after 365 nm or 530 nm irradiation reveal photoswitchable resistor and diode behaviours. The mean current levels are shown with 95% confidence intervals (*N* = 3 separate pores). **b**, The *I*–*V* responses of ensembles of (E111C-pzMe)_7_ recorded after irradiation at four wavelengths (365 nm, 405 nm, 455 nm and 530 nm) and normalized according to the number of pores. Each pore was assumed to contribute +64 pA at +100 mV after 365 nm irradiation. The number of pores in each experiment ranged from 60 to 904 (*N* = 7 experiments). The mean current levels are shown with 95% confidence intervals. **c**, Voltage and wavelength dependences of the per cent gated current (*I*_gate%_ = (1 − *I*_OFF_/*I*_ON_) × 100%), where *I*_ON_ is the current after 365 nm irradiation and *I*_OFF_ is the current at the wavelength of interest. The error bands are 95% confidence intervals. The right schematic shows estimated distributions of intermediate states at PSS for four wavelengths. **d**, The voltage-dependent rectification ratio (*I*_−_/*I*_+_, the ratio of currents recorded under the same magnitude of potential in the opposite polarity) of a single or multiple (E111C-pzMe)_7_ photopores (*N* = 3 experiments; data are mean *I*_−_/*I*_+_ with s.d. shown as error bars). **e**, Current passing through an ensemble of (E111C-pzMe)_7_ photopores in response to an alternating potential. The curves from the top to bottom are the applied voltage and the current responses at 365 nm and at 530 nm. **f**, A truth table for NAND logic achieved using photopores. High and low current levels are achieved by combinations of the input wavelength and the applied potential. The *I*–*V* curves were recorded with a 5 kHz in-line Bessel filter at 25 kHz sampling frequency. A 20 Hz digital Bessel filter was used for data analysis. Recording conditions: 2 M KCl, 10 mM Tris–HCl, 0.1 mM EDTA, pH 8.5, 24 ± 1 °C. Source data.

The ON–OFF behaviour of the (E111C-pzMe)_7_ photopore was further demonstrated over a broader range of applied potentials by ramping the voltage from −200 mV to +200 mV in 10 mV steps. We characterized the current–voltage (*I*–*V*) behaviour of a single (E111C-pzMe)_7_ in the dark after reaching the ON state at 365 nm or the OFF state at 530 nm (Fig. 3a). After irradiation at 530 nm, the photopore exhibited a diode-like non-linear *I*–*V* response; almost no current passed through the pore at positive potentials (0 to +200 mV, *V*_m_ applied to *trans*; Fig. 1f), whereas the current demonstrated resistor-like behaviour at negative potentials (*G* = *I*/*V* = 0.32 ± 0.11 nS at −100 mV, *N* = 3 pores) (Fig. 3a). After irradiation at 365 nm, a less obvious rectification was observed (*G* = *I*/*V* = 0.46 ± 0.06 nS at +100 mV and 0.58 ± 0.12 nS at −100 mV, *N* = 3 pores) (Fig. 3a). In other words, the photopore functioned as a photoresistor after irradiation at 365 nm and as an iontronic diode after irradiation at 530 nm. We speculate that the voltage-dependent behaviour of (E111C-pzMe)_7_ photopores arises from the biased orientation of photoswitches confined within an asymmetric nanopore. The orientation of these charge-neutral photoswitches could be influenced by electro-osmotic flow, which is directionally dependent on the applied voltage. Depending on their orientations, the seven photoswitches could adopt various configurations promoted by the surrounding protein environment, thereby altering the ionic flow.

The dual-mode feature (that is, diode and photoresistor) demonstrated with single pores was reproduced with hundreds of pores in ensemble experiments (*N* = 8 experiments, 100–600 pores per experiment) under 365 nm and 530 nm irradiation (Fig. 3b). Irradiation at 405 nm and 455 nm was also tested; these wavelengths generated current levels between those produced by 365 nm and 530 nm at all applied potentials (Fig. 3b). To give an overview of the voltage-dependent behaviour in the ensemble, the *I*_gate%_ was calculated following *I*_gate%_ = (1 − *I*_OFF_/*I*_ON_) × 100%, where the current level after 365 nm irradiation was *I*_ON_ and that after 405 nm, 455 nm or 530 nm was *I*_OFF_ (that is, *I*_gate%(405 nm)_ = (1 − *I*_405_/*I*_365_) × 100%) (Fig. 3c). Between −150 mV and +100 mV, *I*_gate%_ increased with decreasing negative potential and increasing positive potential for all three wavelengths. The difference in PSS ratios associated with irradiation wavelengths was the primary reason for the wavelength dependence of *I*_gate%_. The rectification ratio of (E111C-pzMe)_7_ (*I*_−_/*I*_+_, the ratio of currents recorded under opposite polarities at the same potential) scaled almost linearly with the amplitude of the applied potential up to ±150 mV (Fig. 3d), and was 5.5 ± 0.7 at ±100 mV in the diode mode after 530 nm irradiation (Fig. 3d).

Nanopore diodes can be used in soft devices as half-wave rectifiers^33^ to convert alternating current to direct current. We tested our photopore as a switchable iontronic diode to effect a unidirectional current across a synthetic bilayer under an alternating voltage at 0.03 Hz (Fig. 3e). After 530 nm irradiation, ~900 (E111C-pzMe)_7_ photopores collectively conducted currents only under a negative potential. After 365 nm irradiation, the current flow replicated the sinusoidal shape of the alternating voltage input, albeit weakly rectified. At higher input frequencies, the capacitive current associated with the bilayer would override the resistive current through the pore, diminishing the rectification. The ionic current output through (E111C-pzMe)_7_ photopores was therefore determined by both the wavelength and the applied potential, resembling the NAND Boolean function. This NAND logic gate leveraged the irradiation wavelength as input 1 (365 nm for 0, 530 nm for 1) and the applied potential as input 2 (−100 mV for 0, +100 mV for 1). A low current output only occurs at +100 mV after irradiation at 530 nm (Fig. 3f).

## Light-to-ionic signal conversion with (E111C-pzMe)_7_

**Fig. 4:**
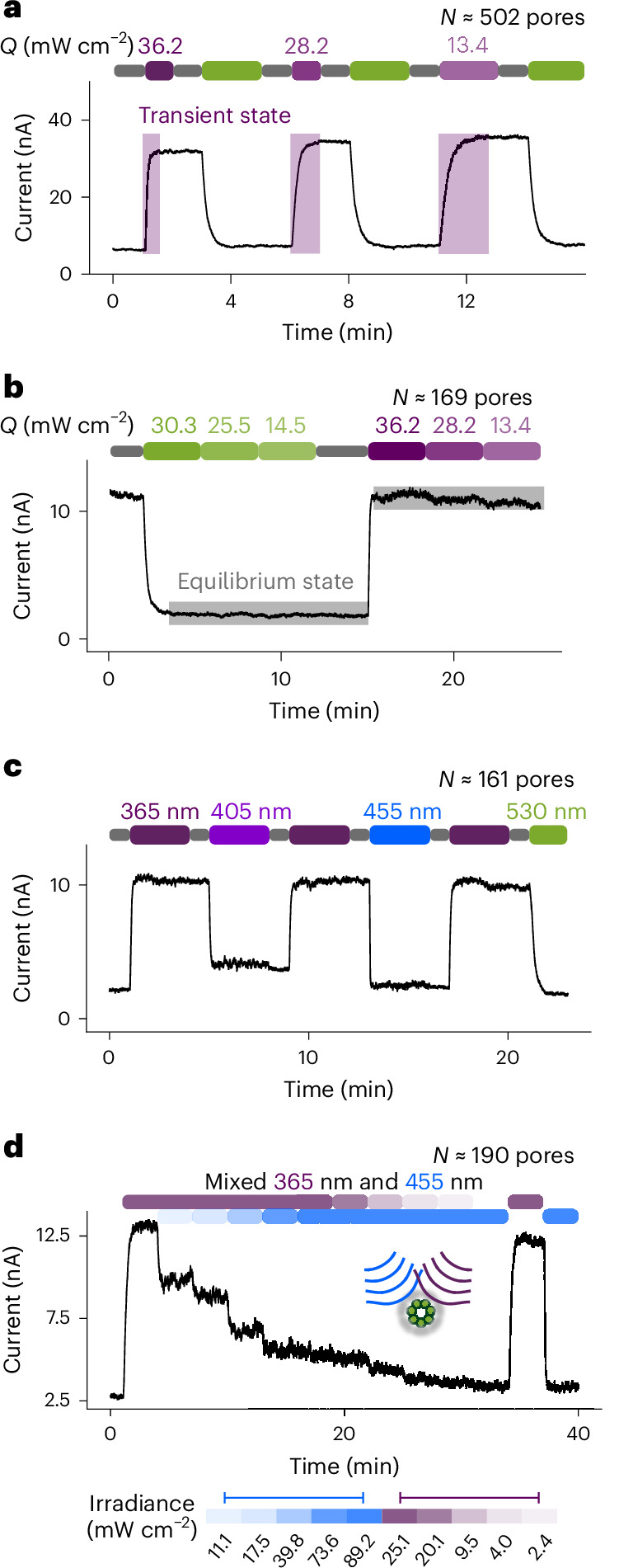
Effects of light intensity and wavelength on the conductance of (E111C-pzMe)_7_ ensembles. **a**, Reduced rate of transition from the low-conductance state (*I*_OFF_) to the high-conductance state (*I*_ON_) due to reductions in the intensity of 365 nm irradiation (purple). The light irradiance (*Q*) is shown above the light sequence. The baseline drift is caused by the degradation of Ag/AgCl electrodes when recording for minutes at the nA level. **b**, Insensitivity of the output current to light intensity after an equilibrium state is reached. When photopores reached equilibrium states of *I*_ON_ = 11.4 nA (530 nm, green) or *I*_OFF_ = 1.8 nA (365 nm, purple), reductions in the light intensity from high to low *Q* did not cause further changes in the current. **c**, At equilibrium, continuous irradiation result in an ON state at 365 nm (purple), partially OFF states at 405 nm (violet) and 455 nm (blue) and a fully OFF state at 530 nm (green). **d**, Intermediate current levels can be accessed by polychromic light. Mixed-wavelength irradiation over 3-min intervals with various ratios of 365 nm and 455 nm light allowed fine control of the PSS ratios with >200 photopores. The current traces were recorded at +100 mV using a 5 kHz in-line Bessel filter at 25 kHz sampling frequency. A 20 Hz digital Bessel filter was used for data analysis. Recording conditions: 2 M KCl, 10 mM Tris–HCl, 0.1 mM EDTA, pH 8.5, 24 ± 1 °C.

The rate of photoisomerization of a photoswitch is typically proportional to the light intensity^5,34,35^. Accordingly, 365 nm irradiation of more than 600 (E111C-pzMe)_7_ pores at 36.2, 28.2 and 13.4 mW cm^−2^ (Supplementary Fig. 7) increased the duration of the OFF–ON transition from 5.8 ± 0.5 s to 7.1 ± 0.6 s, and then to 11.4 ± 2.6 s (*N* = 4 ensembles; Fig. 4a). After the equilibrium current level was reached under a given wavelength of irradiation, reducing the light intensity led to no current change (Fig. 4b). In contrast, common electronic components and optogenetic tools often exhibit transient current responses that vary with light intensity.

An ionic signal conducted by multiple (E111C-pzMe)_7_ photopores at an intermediate current level between *I*_ON_ and *I*_OFF_ could be accessed by switching off the irradiation immediately after reaching the desired current level during an ON–OFF transition at a given wavelength. However, it was challenging to reproducibly capture a transient current level of interest during rapid ON–OFF transitions. An alternative strategy was devised using polychromic light to produce equilibrium current levels as determined by the PSS of photoswitches (Fig. 4c,d), which would be unattainable by continuous irradiation at either one of the wavelengths. Light from 365 nm and 455 nm LEDs was combined through a fibre coupler. The graded modulation of ionic signal was demonstrated by varying the relative intensity of the 365 nm and 455 nm irradiation projected onto the multiple (E111C-pzMe)_7_ pores in 3 min steps. Ionic signal levels between those recorded at PSS_365_ and PSS_455_ were obtained (Fig. 4d). This approach allowed for finer control over the graded modulation of ionic current through (E111C-pzMe)_7_ photopores.

**Fig. 5:**
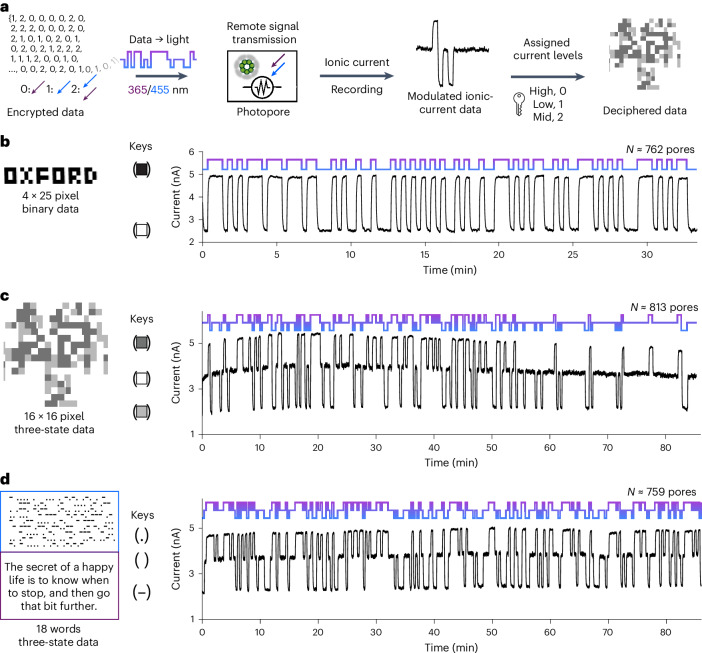
Light-to-current signal conversion. **a**, An overview of the process. Image or text data were encoded as light sequences that produced ionic current responses from (E111C-pzMe)_7_. By using pre-determined keys to assign the current levels, the ionic currents were deciphered to reconstruct the original data. **b**, Transmission of a binary pixel pattern with monochromic 365 nm and 455 nm irradiation. **c**, Transmission of a three-state pixel art in a light sequence. The image of the αHL pore was generated from PDB 7AHL and converted to three-colour pixel art. Each pixel was assigned to 0, 1 or 2 based on the colour. The matrix of pixels was flattened to 1D to produce the light sequence. **d**, Transmission of three-state Morse code. The blue box is the Morse code from the deciphered current trace and the purple box is the translated text. The light sequence was the input at wavelengths of 365 nm, 455 nm or mixed 365/455 nm at a rate of 20 s per bit to generate a current pattern consisting of three levels. The current traces were recorded at +100 mV using a 5 kHz in-line Bessel filter at 25 kHz sampling frequency. A 20 Hz digital Bessel filter was used for data analysis. Currents from (E111C-pzMe)_7_ ensembles were recorded in 150 mM KCl, 20 mM Tris–HCl, 0.1 mM EDTA, pH 8.5, 24 ± 1 °C.

Inspired by the slow thermal relaxation of arylazopyrazole and the graded modulation of the ionic signal, we investigated light-to-ionic signal conversion by an ensemble of iontronic photopores. We first encrypted information-bearing binary data within light sequences that alternated between 365 nm and 455 nm radiation (Fig. 5a). Functioning as a light-to-current converter, the (E111C-pzMe)_7_ photopores translated the light sequences into two-level current patterns (Fig. 5a), as illustrated by a representative segment of a trace with an input rate of 20 s per bit (Fig. 5b). Higher input rates were achieved with increased light intensities, reaching 1.5 s per bit at 365 nm and 1.9 s per bit at 455 nm using the maximum LED outputs. Over a 90 min experiment, no photobleaching was observed. By associating the ON state with a black pixel and the OFF state with a white pixel, a pixel art spelling ‘OXFORD’ was reconstructed from a binary light message. Furthermore, the addition of mixed irradiation at both 365 nm (36.2 mW cm^−2^) and 455 nm (56.3 mW cm^−2^) expanded the alphabet to a three-state system, which was applied to demodulate a pixel art representation of a protein nanopore and a text message written in Morse code (Fig. 5c,d). In principle, by using mixed-wavelength irradiation, the (E111C-pzMe)_7_ photopores could output a series of current levels, between the levels at PSS_365_ and PSS_530_, as the basis of complex encoding systems. With our equipment, the current levels fluctuate ±0.3% in the dark after a 20 Hz digital filter, and the *I*_gate%_ was ~0–85%. Thus, an upper-level estimate for the number of assignable levels would be ~300.

## Conclusions

In this work, we constructed protein nanopores with unitary conductance values that changed under light control. Among the tested photopores, we achieved quantitative, prolonged and reversible modulation of ionic currents carried by (E111C-pzMe)_7_ pores following irradiation at 365 nm (ON, high conductance) and 530 nm (OFF, low conductance). Notably, when an ON state (E111C-pzMe)_7_ was switched OFF at +100 mV, the unitary conductance reduced by >95%. Similarly, a current reduction of >85% occurred when multiple (E111C-pzMe)_7_ pores were used in ensemble experiments. This sustained ON state and the almost complete OFF state are highly advantageous for real-time control over transmembrane ionic communication in nanotechnology or synthetic biology applications.

The photopores described here differ from previously described photoregulated nanopores in two aspects. First, the photoswitches controlled the conductance of a fully assembled nanopore reversibly, rather than the process of irreversible nanopore assembly^27^. Second, the current–voltage characteristics of (E111C-pzMe)_7_ exhibited two switchable states: a resistor-like ON state, after 365 nm irradiation, and a diode-like OFF state, after 530 nm irradiation. In the diode mode, there is no delay in the rectification of (E111C-pzMe)_7_ after the reversal of the polarity of the applied potential, in contrast to the previous (non-photoswitchable) arginine-rich αHL diode^33^.

One impact of photopores in the field of nanoscale iontronics might be in light-to-ionic current signalling. In the case of the (E111C-pzMe)_7_ pore, we have used the ability to produce multiple output current levels, such as the three-state system (blue, mixed and UV), to demonstrate the ability to reconstruct images or text encoded in light pulses. It follows that by hosting light-modulated nanopores in a two-dimensional array of lipid bilayers, an artificial retina might be created to detect coloured images. Our previous work on droplet-hydrogel bilayer biopixels reported a related approach for monochromic 530 nm light^36^.

The photopores might also be incorporated into three-dimensional-printed droplet-based synthetic tissues where the spatiotemporal control of communication between compartments is key to the control of tissue-like behaviour^37,38^. There, they could be used, among other things, to trigger the release of ions or small molecules^38^, mediate shape changes^37^ or produce motion^39^. We further envisage the development of additional photopores by the conjugation protocol reported here to gain control over additional properties such as selectivity in the transport of small molecules and the ability to mediate the translocation of biopolymers.

## Methods

### Conjugation of photoswitches

Br-pzMe, Br-pzH or Br-fAzo (the photoswitches) were dissolved at 3 mM in dimethylsulfoxide (DMSO). Purified monomers of the αHL cysteine mutants were buffer exchanged and diluted to 0.5 mg ml^−1^ in low TE buffer (10 mM Tris–HCl, pH 8.0, and 0.1 mM EDTA). A photoswitch was added to a monomer solution stepwise over 18 h to a final concentration of 750 µM at 20 °C. The extent of reaction was assessed by liquid chromatography–mass spectrometry. The conjugated protein solution was passed through a desalting spin column (Thermo Fisher) equilibrated in the low TE buffer to remove excess photoswitch and the eluent was stored at −80 °C.

### Single-channel and ensemble experiments

The aperture in the Teflon partition of the recording chamber was pre-treated with hexadecane (~5 µl, 1% in pentane). After 10 min, recording buffer (1 ml, 10 mM Tris–HCl pH 8.5, 2 M KCl and 0.1 mM EDTA) was added to both the *cis* and *trans* compartments (Supplementary Fig. 7). A lipid bilayer was formed on the aperture by using the Müller–Montal method with 1,2-diphytanoyl-3-*sn*-phosphatidylcholine (Avanti Polar Lipids). A pair of Ag/AgCl electrodes was placed in each compartment through a salt bridge (3 M KCl in 2% (w/v) agarose). The electrodes were covered with black tape to avoid exposure to light from the LED.

To insert a single pore into the bilayer (Supplementary Fig. 10a), homoheptamer (0.1 µl, 0.2 mg ml^−1^) was added to the *cis* compartment. Once a pore had inserted and showed a photoresponse, the buffer was perfused five times to prevent further insertions. To achieve the stepwise insertion of multiple pores, more homoheptamer (10 µl, 0.2 mg ml^−1^) was used (Supplementary Fig. 9a).

The characterization of the photopore ensemble was recorded using photopore monomers pre-irradiated at 530 nm for 30 min before use to ensure that the monomers were in the *E* state prior to assembly (Supplementary Fig. 10c). The monomers (~5 µl, ~0.2–0.5 mg ml^−1^) were added to the *cis* compartment while the 530 nm LED was switched on. Pore insertion occurred under an alternating applied potential of ±20 mV, and the insertion rate slowed down over 15 min. Excess monomers were removed by perfusion of the *cis* compartment.

Ionic currents were recorded through Ag/AgCl electrodes connected to a patch clamp amplifier (Axopatch 200B, Axon Instruments). The signal was filtered with an in-line four-pole low-pass Bessel filter (80 dB per decade, 5 kHz). A Digidata 1322 A digitizer (Molecular Devices) was used to convert the analogue signal to digital form. The data were analysed with the pCLAMP 10.3 software suite (Molecular Devices). Electrical traces were plotted with Python (3.8.8), Pyabf (2.3.5), Matplotlib (3.3.4) and Seaborn (0.11.1).

### Transmembrane signal transmission by light-to-current conversion

To achieve the light-to-current signal conversion, three steps are required. First, the encrypted digital data, as pixel art or text, were converted into a monochromatic or polychromatic one-dimensional (1D) light sequence. In the second step, the 1D light sequence was sent to an Arduino UNO microcontroller to trigger the 365 nm and 455 nm LEDs accordingly. Due to the switching rate of the photopore, each unit of irradiation (a bit) was optimized to last for 20 s to produce rectangular signals. The final step was to process the electrical recording to visualize and analyse the output signal. The electrical recording trace was processed in reverse from the current signal to give the pixel image or text.

### Statistics and reproducibility

Quantitative analysis related to photopore currents was performed with a minimum of three replicates. For the single-molecule experiment, each replicate means an individual nanopore. For the ensemble experiment, each replicate refers to a lipid bilayer with multiple pores. No statistical method was used to pre-determine sample size. No data were excluded from the analyses.

## Online content

Any methods, additional references, Nature Portfolio reporting summaries, source data, extended data, supplementary information, acknowledgements, peer review information; details of author contributions and competing interests; and statements of data and code availability are available at 10.1038/s41565-024-01823-x.

## Supplementary information

Supplementary Information Supplementary Figs. 1–33 and synthesis of Br-pzMe, Br-pzH and Br-fAzo.

## Source data

Source Data Fig. 1 Source data for the UV–vis spectra of Br-pzMe.

Source Data Fig. 2. Source data for the photopore gating efficiency.

Source Data Fig. 3 Source data for *I*–*V* response, gating efficiency and rectification.

## Data Availability

Electrical recording source data are available from the corresponding authors upon request. Source data are provided with this paper.
